# INVESTIGATING ALTERNATIVES TO THE FISH EARLY-LIFE STAGE TEST: A STRATEGY FOR DISCOVERING AND ANNOTATING ADVERSE OUTCOME PATHWAYS FOR EARLY FISH DEVELOPMENT

**DOI:** 10.1002/etc.2403

**Published:** 2013-10-01

**Authors:** Daniel Villeneuve, David C Volz, Michelle R Embry, Gerald T Ankley, Scott E Belanger, Marc Léonard, Kristin Schirmer, Robert Tanguay, Lisa Truong, Leah Wehmas

**Affiliations:** † US Environmental Protection AgencyDuluth, Minnesota, USA; ‡ Arnold School of Public Health, University of South CarolinaColumbia, South Carolina, USA; § International Life Sciences Institute, Health and Environmental Sciences InstituteWashington, DC, USA; ‖ Procter & GambleCincinnati, Ohio, USA; # Research and Innovation, L'OréalAulnay-sous-Bois, France; †† Eawag, Swiss Federal Institute of Aquatic Science and Technology, Dübendorf; EPF Lausanne, School of Architecture, Civil and Environmental EngineeringLausanne; ETH Zürich, Institute of Biogeochemistry and Pollutant DynamicsZürich, Switzerland; ‡‡ Oregon State UniversityCorvallis, Oregon, USA

**Keywords:** Adverse outcome pathways, Aquatic toxicology, Risk assessment, Mode of action, Swim bladder, Fish early-life stage toxicity, Animal alternative

## Abstract

The fish early-life stage (FELS) test (Organisation for Economic Co-operation and Development [OECD] test guideline 210) is the primary test used internationally to estimate chronic fish toxicity in support of ecological risk assessments and chemical management programs. As part of an ongoing effort to develop efficient and cost-effective alternatives to the FELS test, there is a need to identify and describe potential adverse outcome pathways (AOPs) relevant to FELS toxicity. To support this endeavor, the authors outline and illustrate an overall strategy for the discovery and annotation of FELS AOPs. Key events represented by major developmental landmarks were organized into a preliminary conceptual model of fish development. Using swim bladder inflation as an example, a weight-of-evidence–based approach was used to support linkage of key molecular initiating events to adverse phenotypic outcomes and reduced young-of-year survival. Based on an iterative approach, the feasibility of using key events as the foundation for expanding a network of plausible linkages and AOP knowledge was explored and, in the process, important knowledge gaps were identified. Given the scope and scale of the task, prioritization of AOP development was recommended and key research objectives were defined relative to factors such as current animal-use restrictions in the European Union and increased demands for fish toxicity data in chemical management programs globally. The example and strategy described are intended to guide collective efforts to define FELS-related AOPs and develop resource-efficient predictive assays that address the toxicological domain of the OECD 210 test. *Environ Toxicol Chem* 2014;33:158–169. © 2013 The Authors. *Environmental Toxicology and Chemistry* published by Wiley Periodicals, Inc. on behalf of SETAC. This is an open access article under the terms of the Creative Commons Attribution License, which permits use, distribution, and reproduction in any medium, provided the original work is properly cited.

## INTRODUCTION

Adverse outcome pathways (AOPs) have been proposed as frameworks to link direct, molecular initiating events to adverse outcomes measured at higher levels of biological organization considered relevant to risk assessment [1]. An AOP generally describes a “sequence of events from the exposure of an individual or population to a chemical substance through an adverse effect at the individual level (for human health) or population level (for ecological health)” [2]. Development of AOPs can facilitate identification of key uncertainties and corresponding research gaps specific to biological mechanisms responsible for perturbation of a system and toxicity. Additionally, once described and annotated, AOPs can be used to aid the identification of alternative, high-throughput predictive assays and testing strategies that can help inform risk assessments. When developed and validated, these alternative assays, along with computational tools for concentration–response extrapolation across multiple levels of biological organization, are intended to provide flexibility in testing based on risk-management needs while reducing animal use and study costs [3,4]. The increased mechanistic understanding provided by an AOP framework will also facilitate extrapolation across species, end points, and chemicals. The term “AOP,” as used in the present study, is synonymous with “mode of action” within the human health risk assessment community to describe a biologically plausible series of key events linking chemical exposure to an adverse health effect [5–7].

The fish early-life stage (FELS) test guideline (Organisation for Economic Co-operation and Development [OECD] test guideline 210) is the most frequently used bioassay for chronic fish toxicity, supporting aquatic ecological risk assessments and chemical management programs around the world. Although valuable for predicting aspects of fish full life-cycle toxicity, FELS tests are labor- and resource-intensive and, for each chemical, require a minimum of 480 fish—excluding breeding stock and animals used for range-finding prior to conducting the guideline test—and 1 to 3 mo from test initiation to termination. While the FELS test emphasizes interpretation of toxicity based on the apical end points of survival and growth, it is further supplemented with information on behavior and developmental abnormalities. Fish early-life stage tests are therefore not designed to provide substantive information about chemical mode of action. Beyond these AOP–oriented points, criticisms have also been raised regarding the statistical power of OECD test guideline 210 even for common apical end points [8]. The development and implementation of alternative testing strategies for screening and prioritizing chemicals have the potential to reduce the cost and number of animals required for estimating FELS toxicity while, at the same time, providing insights into chemical modes of action that can lead to improved prediction of toxicity outcomes based on chemical properties and structure.

**Figure 1:**
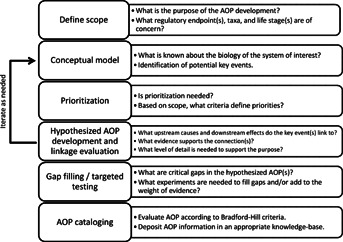
Overview of a generalized strategy for adverse outcome pathway (AOP) discovery and development. Although the process is depicted as linear, iterations between various steps, particularly conceptual model development and hypothesized AOP development/linkage evaluation, may be needed.

As part of an ongoing effort to develop alternatives to the FELS test, the International Life Sciences Institute's Health and Environmental Sciences Institute's (HESI) Animal Alternatives in Environmental Risk Assessment Technical Committee is actively engaged with multiple partners to identify and develop AOP knowledge relevant to FELS. The focus on development of FELS-related AOPs evolved from discussions and consensus prior to and during a HESI-sponsored workshop held in June 2010 and a tiered testing strategy subsequently proposed by Volz et al. [9]. The present study synthesizes ideas and discussion from a follow-up HESI-sponsored expert workshop held in May 2012 and is based on the intellectual contributions of workshop participants (see *Acknowledgment*). The aim of the present study was to guide collective efforts to define FELS–related AOPs and support development of resource-efficient predictive assays that address the toxicological domain of OECD test guideline 210. We outline a generalized approach to AOP discovery and development (Figure 1) and illustrate its application for supporting the development of alternatives to OECD test guideline 210. Additionally, we define key research objectives for moving beyond qualitative AOP descriptions to quantitative application of alternative testing in hazard and risk assessment.

## DEFINE SCOPE

An important first step in any AOP development project is to define the scope of the activity (Figure 1). What is the purpose for AOP development? What toxicological end points are of concern? What taxonomic group(s) and life stage(s) are of interest? And, perhaps equally important, what is beyond the scope of the effort? This scoping process defines an initial set of boundaries for the AOP development activity and provides insight into the level of resources that will be required to support it. Depending on the needs of the program, investigator, or scientific community, the scope may be broad (e.g., those for which the goal is to connect a broad range of potential molecular initiating events to a specific apical end point of interest) or narrow (e.g., those defined by linkage of a particular molecular initiating event to a specific apical end point in a single species).

The scope of AOP development defined for the HESI-coordinated Animal Alternatives in Environmental Risk Assessment project has been relatively broad in scale. The current priority is to support development of cost-effective alternatives to the FELS test (OECD 210). Consequently, the scope for this activity is largely defined by the existing test guideline [10]. The primary apical end points of regulatory interest in the test are growth and survival. Data on morphological or behavioral abnormalities may be collected and are of interest but only to the extent that these end points are linked to adverse impacts on growth or probability of survival. The test guideline only requires that fish be exposed immediately following fertilization until independent feeding. In practice, however, the recommended test durations range from approximately 30 d to 90 d for the most commonly tested species [8]. This is partly driven by the fact that survival to independent feeding alone has no intrinsic value to population sustainability. In principle, the FELS test is intended to be predictive of outcomes that would occur in a fish full life-cycle test, based on the assumption that early life stages are generally the most sensitive for many chemicals [11–14]. Therefore, in a population context, the probability of young-of-year survival or survival to reproductive maturity is arguably the relevant outcome of regulatory interest, which should serve as an anchor for all AOPs developed as part of this effort.

In terms of taxa, the test guideline does not specifically preclude any classes of fish. In practice, however, all “recommended” and “well-documented” species covered in the test guideline are of the infraclass Teleostei. Therefore, for the purposes of the HESI effort, the primary emphasis is on the development of AOPs for teleosts. In the near term, the effort is not concerned with, for example, agnathans, chondrichthyans, or lungfishes (Dipneustei), which exhibit some significant differences relative to developmental landmarks (e.g., jaw development, ossification, stomach, and gas exchange structures). The relevant scientific and toxicological literature is strongly biased toward a relatively small number of species, notably those for which development has been described in detail (e.g., zebrafish [*Danio rerio*]) as well as those that have been used extensively in toxicity testing (e.g., zebrafish, fathead minnow [*Pimephales promelas*], Japanese medaka [*Oryzia latipes*], and rainbow trout [*Oncorhynchus mykiss*]). Nonetheless, based on the scope of OECD test guideline 210 and its intended regulatory application, it is recognized that efforts should be made to develop AOPs that are generalizable within the teleost infraclass and to the extent possible to ray-finned fish (Actinopterygii), annotating key events or links accordingly if there are known limitations to that generalizability.

Finally, OECD test guideline 210 is relatively agnostic to how the adverse outcome occurs. While it does recommend some intermediate observations that could be collected (e.g., time to start and end of hatching, length and weight of surviving animals, abnormal behaviors), these end points do not provide adequate resolution to define specific potential molecular initiating events to consider in, or exclude from, the AOP development effort. Therefore, despite a well-defined anchor to young-of-year survival on the apical end and a focus on teleost fish, the scope of AOP development is quite broad at the molecular level. Since listing all possible molecular initiating events is impractical, the scope dictates that AOP development be organized around intermediate key events in the pathways, which then could be linked with young-of-year survival and traced back to relevant initiating events. Thus, rather than take either a bottom–up approach (molecular initiating event to adverse outcome) or a top–down approach (adverse outcome to molecular initiating event) to construction of hypothesized AOPs, a middle–out approach (organ to adverse outcome and molecular initiating event) seemed most appropriate and practical for the scope of our current effort. This middle–out approach allows for anchoring to organ-level phenotypes important for early fish development (and presumably early vertebrate development).

## CONCEPTUAL MODEL

The scope of an AOP development project sets the bounds of the relevant biological system(s) to be considered. Once those bounds are defined, a useful next step in AOP development is to outline what is known about the function of that biological system in the form of a conceptual model (Figure 1). Fundamental understanding of the normal regulation of the biological system(s) of interest provides the foundation for understanding how perturbation of different elements of that system will impact overall function. The exact nature and level of detail to include in the conceptual model are scalable and depend on scope. Nonetheless, regardless of the scope of AOP development, the objective of the conceptual model is to aid in the identification of “key events” relevant to the AOP(s) of interest. In the context of AOP development, those key events are represented as nodes in an AOP diagram. Key events have 2 important characteristics [5,6]: 1) they have to be measurable or observable, and 2) they should be necessary to functions whose disruption can be causally linked to the adverse outcome (through either direct evidence or biological plausibility). For FELS tests, that would mean biological events necessary for growth and survival at least to independent feeding and transition to juvenile fish, if not to adulthood.

An example of a conceptual model development was provided by Ankley et al. [15]. In that instance, important aspects of the biology regulating fish reproduction were organized into a multicompartmental model. The model was then used to hypothesize specific targets (i.e., molecular initiating events) along the hypothalamic–pituitary–gonadal axis through which chemicals could potentially perturb normal reproductive functions to cause reproductive toxicity in fish. The model also served to inform the identification of end points or measurable intermediate events that could be used to test hypothesized AOPs.

**Figure 2:**
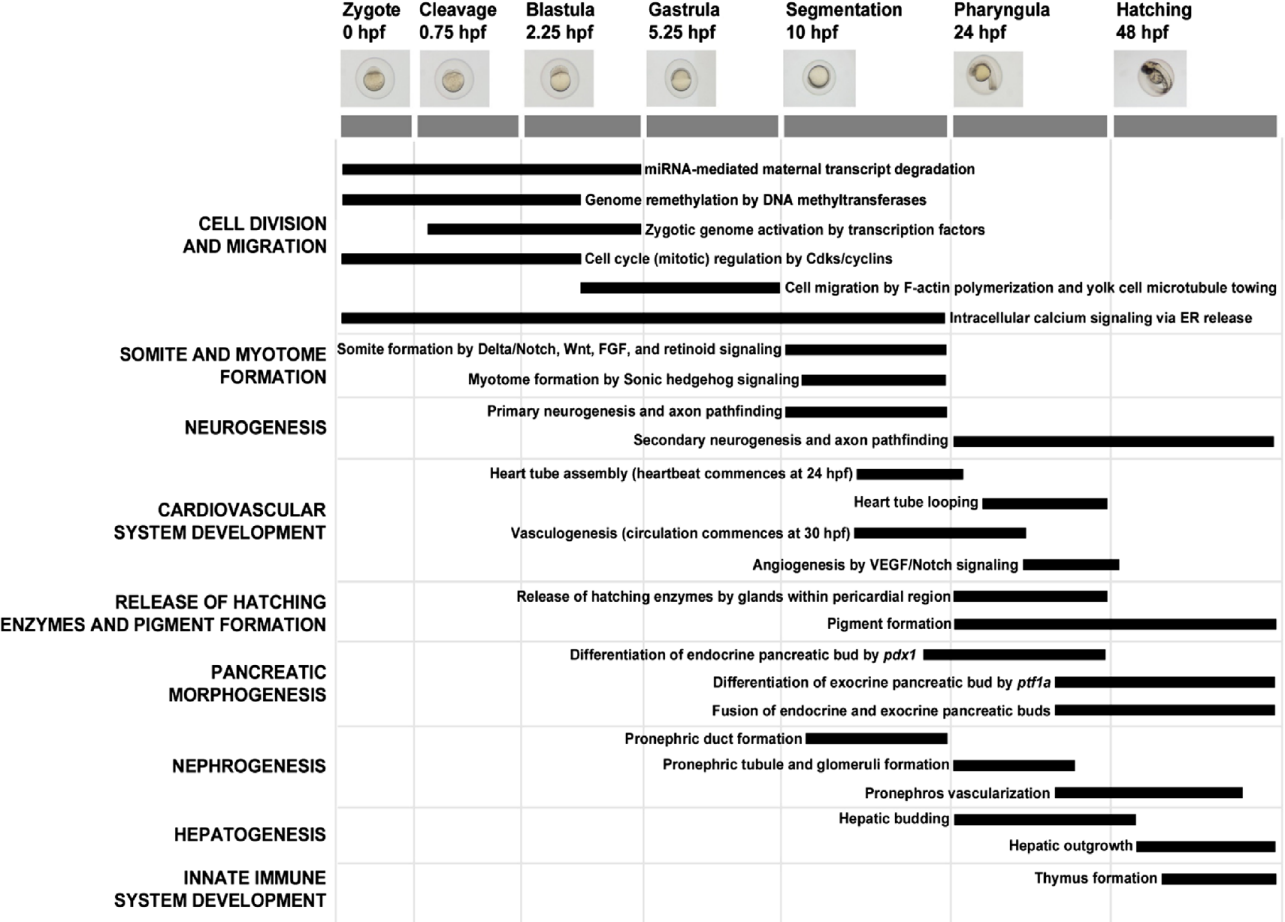
Preliminary conceptual model of developmental landmarks during zebrafish embryogenesis. The *y*-axis lists a sampling of major developmental landmarks, and the *x*-axis shows the timing and stages of zebrafish embryogenesis. Black bars denote the approximate duration of biological events that underlie each developmental landmark. hpf = hours postfertilization.

The challenge in defining AOPs relevant to FELS toxicity differs from this previous example in several ways. First, because the apical outcomes of interest for FELS are growth and survival, all organs and organ systems are potentially involved. Second, because the life stage of interest is the developing organism, specifically a highly dynamic period from before cleavage of the blastodisc through hatching and attainment of a well-developed juvenile form, the structure of the system is both complex and dynamic. Identification of critical periods of developmental susceptibility has long been a unifying concept in human and environmental safety assessment [11,16]. However, the complexity of the processes involved does not lend itself to the same type of compartmentalized conceptual modeling approach employed by Ankley et al. [15]. Instead, a conceptual model organized around a series of developmental, morphological landmarks arranged along a relative temporal scale is more appropriate (Figure 2). Examples of landmarks include development of the central nervous system, cardiovascular system, liver, and kidney. Because these are morphological landmarks, they are by definition observable and thus, meet the first central criterion of key events. After assembling the conceptual model, initial prioritization of events depicted as targets for subsequent AOP development could be made by evaluating the 2nd criterion, that is, whether the developmental landmark is plausibly linked with the ability to grow and survive to adulthood. Most directly, this invokes the ability to survive in a controlled laboratory environment. However, since it is populations in the environment, not those in the laboratory, that we aim to protect, any plausible linkage(s) to survival in the field where the fish must compete for limited food and habitat and avoid predation should also be considered. Given these criteria, landmarks such as cell division, somite formation, cardiac development, and liver formation can be readily identified as relevant key events. The relevance of other developmental landmarks, such as pigment formation, to adverse population-level impacts is less clear. Nonetheless, the conceptual model provides a framework of biological context from which to begin hypothesizing AOPs.

## PRIORITIZATION

As noted above, the scope for this AOP development activity is relatively broad. It is clear that comprehensively addressing this scope will require wide-ranging expertise and a coordinated effort over a significant period. Given the likelihood that the full scope of AOPs relevant to FELS testing could take years to develop in detail, an important consideration is how to most effectively prioritize the AOP development effort (Figure 1).

The need to improve the efficiency and cost-effectiveness of toxicity testing along with a societal desire to reduce the use of animals in testing, particularly in the European Union, are key drivers of the interest in developing alternatives to OECD test guideline 210. In recent years, it has been demonstrated that fish embryos are amenable to high-throughput testing approaches [17,18]. Though definitions of protected and nonprotected stages of fish vary, current legislation within the European Union indicates that fish embryos (prehatch stages) and eleuthereoembryos through the onset of independent feeding (e.g., 5–6 d postfertilization for small fish models such as zebrafish or fathead minnows) are nonprotected life stages [19,20]. Belanger et al. [21] identified a conservative estimate (small proportion of the population) for the onset of exogenous feeding in zebrafish to occur around 96 h. More recently, the European Commission officially announced that protection will be afforded to fish (*D. rerio*) at 5 d postfertilization at 28 °C of culturing [22,23]. Other regulatory jurisdictions do not clearly distinguish protected and nonprotected stages of fish but do provide indications based on acceptance of certain assays or through the encouragement of new assays and approaches (e.g., Japan and the United States). Therefore, expanded (in terms of end points measured and data collected) and optimized versions of a fish embryo test [24] have been proposed as potential near-term alternatives to FELS testing [9,19].

**Figure 3:**
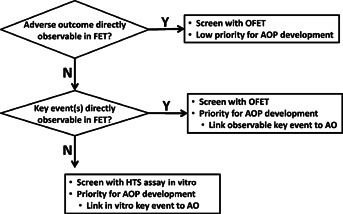
Prioritization framework for development of alternatives to the fish early life stage toxicity test (Organisation for Economic Co-operation and Development test guideline 210) and associated adverse outcome pathways (AOPs). HTS = high-throughput screening; FET = fish embryo test; OFET = optimized fish embryo test; AO = adverse outcome, which for the purposes of the present study refers to reduced probability of young-of-year survival.

Identification of an optimized fish embryo test as an available near-term alternative to the FELS test provided a means to prioritize the scope of AOP development (Figure 3). For example, highest priority could be assigned for AOPs that have key events that could plausibly be observed over the typical span of a fish embryo test (e.g., within 96 h postfertilization for zebrafish) but for which impacts on growth and/or survival likely would not manifest until after hatch and independent feeding (i.e., after the duration covered by the embryo test). Potential examples include craniofacial malformations leading to impaired jaw motion or mouth opening or impaired fin formation. Likewise, incorporation of high-content quantitative imaging of cardiovascular development, neurogenesis, and/or gill development, all key events in AOPs, into an optimized fish embryo test could support predictive hazard screening, while obviating the need to screen chemicals in several additional molecular screening assays (e.g., aryl hydrocarbon receptor activation, acetylcholinesterase inhibition, and gill cell cytotoxicity as proposed by Volz et al. [9]). Similarly, functional assessments predictive of later-stage cardiotoxicity and neurotoxicity outcomes such as larval heart rates and behavior (locomotor activity and photomotor response) could be rapidly quantified within developing zebrafish embryos [25–29]. In each case, development of AOPs would serve to define the predictive linkages between end points that could be incorporated into an optimized fish embryo test and probable young-of-year mortality.

In contrast, development of AOPs centered on key events whose perturbation would be expected to cause mortality prior to hatching could be given lowest priority (Figure 3), because the apical outcome of impaired survival could still be directly observed in the alternative test. In such cases, no predictive linkage is needed. Examples of this low-priority group include disrupted cell division and migration or impaired somite formation, both of which could be expected to cause embryo mortality prior to hatch.

Intermediate or high priority could be assigned to development of AOPs where mortality would not occur prior to the onset of exogenous feeding and whose key events could not be observed in the context of an optimized embryo test (Figure 3). This could include, for example, certain impairments in immune system development or defects in sensory structures required to capture food or avoid predation. Additional molecular screening assays with complementary AOPs would need to be developed to predict outcomes in such cases. Overall, it was concluded that, in the near term, a priority should be development of AOPs that would support the design of an optimized fish embryo test that was both high-throughput and high-content in terms of providing data concerning key events that are predictive of longer-term adverse outcomes. This pragmatic prioritization can be viewed as a refinement to scope aimed at making the task more manageable and maximizing the utility of the resources available for AOP development.

## HYPOTHESIZED AOP DEVELOPMENT AND LINKAGE EVALUATION

A sense of the pragmatic priorities, along with a conceptual model outlining relevant background knowledge and functional relationships between important components of a biological system or, in this case, developmental events, serves as an important reference point for AOP development. However, the most critical aspect of AOP development is defining the series of plausible and scientifically defensible linkages between the molecular initiating event(s), the intermediate key events, and the adverse outcome of interest. As stated in the recently developed OECD guidance document on developing and assessing AOPs [30], this entails not only identifying the relevant series of key events but also outlining and evaluating the scientific evidence supporting the linkages between the key events. In particular, application of the Bradford-Hill criteria for causation has been recommended as a means for evaluating the weight of evidence supporting the linkages in an AOP or mode of action [6,31]. At the same time, evaluating the plausibility and supporting evidence for the linkages also helps identify gaps and uncertainties in the AOPs. Gaps and uncertainties help define the confidence with which the AOPs can be used to support predictive toxicology, as well as areas where research is needed to support or reject those potential predictive relationships. We feel that the process of hypothesized AOP development and linkage evaluation (Figure 1) is perhaps best illustrated with an example such as the one provided below.

### Swim bladder inflation as a case example

Swim bladder inflation is an example of a developmental landmark that generally occurs during eleuthereoembryogenesis, a stage when the yolk sac is depleted and fish initiate independent feeding [32–34]. In transparent larval fish, swim bladder inflation can be observed directly by light microscopy [35]. It can also be detected based on swimming performance along with orientation and position in the water column, with many species sinking to the bottom when unable to inflate their swim bladder [36–38]. Because it is easily observable, swim bladder inflation meets the first criterion defining key events in an AOP.

**Figure 4:**
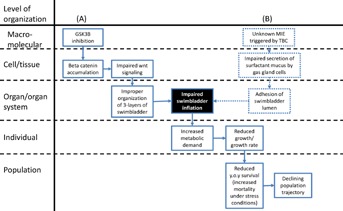
Two examples of hypothesized adverse outcome pathways (AOPs) for the key event of impaired swim bladder inflation (black background). (**A**) Hypothesized AOP linking glycogen synthase kinase 3β inhibition to reduced young-of-year (y.o.y) survival in fish via impacts on swim bladder inflation; (**B**) hypothesized AOP linking impaired gas gland cell mucus secretion to reduced young-of-year survival via impacts on swim bladder inflation. GSK3B = glycogen synthase kinase 3β; MIE = molecular initiating event; TBC = tris(2,3-dibromopropyl)isocyanurate.

Swim bladder inflation is also important for growth and survival, 2 apical end points traditionally considered relevant to risk assessment. Failure of the swim bladder to inflate is not, by itself, lethal to the organism. Fish with noninflated swim bladders can survive both under aquaculture conditions and in natural habitats [38,39]. The probability of their survival is greatly diminished, however, particularly in natural habitats where food resources are limited and energy must be expended on predator avoidance, diel migrations to access food, and so on. [38]. In a study on natural populations of yellow perch (*Perca flavescens*), Czesny et al. [37] demonstrated that failed swim bladder inflation significantly increased the likelihood of young-of-year mortality. This was attributed to increased metabolic demand associated with loss of neutral buoyancy and the corresponding need to expend greater energy to maintain position in the water column [37]. As one might expect, increased metabolic demand associated with a lack of swim bladder inflation also resulted in reduced growth, which in turn was associated with increased vulnerability to predation and reduced efficiency in capturing evasive prey [37]. Consistent with this observation in the field, reduced growth rates in larvae with noninflated swim bladders have been reported under aquaculture conditions as well [38]. Overall, in terms of both biological plausibility and evidence in the literature, a strong case can be made that swim bladder inflation is relevant to both growth and survival (Figure 4). Although failed swim bladder inflation may not result in mortality during the OECD 210 test, this partial AOP and available weight of evidence support the relevance of impaired growth as a predictor of reduced young-of-year survival.

For the purpose of developing viable alternatives to the FELS test, further AOP development upstream (i.e., in the direction of a target molecular initiating event) is needed. As noted above, swim bladder inflation generally occurs around the same time as the onset of feeding. Therefore, direct observation of the key event of swim bladder inflation could not be reliably incorporated into a fish embryo test designed to end before the onset of independent feeding. Likewise, the effect on growth would not be evident until sometime after the yolk reserves were depleted, requiring the fish to forage for food. Therefore, either an alternative predictive end point suitable for incorporation into an embryo test or a complementary assay (or multiple complementary assays) would be needed to effectively screen chemicals as potential early-life stage disruptors of swim bladder inflation or function, in lieu of conducting a longer-term FELS test.

Connecting the key event of failed swim bladder inflation to the relevant molecular initiating event(s) involves developing a more thorough understanding of biological processes than described in the initial conceptual model focused on morphological landmarks during FELS development. In general, as hypothesized AOPs develop and expand, particularly moving from one level of biological organization to another, iterations of conceptual model development, key event identification, and linkage evaluation will often be required (Figure 1). The exact number of iterations is dictated by the purpose of AOP development and the respective level of biological detail needed to confidently make predictions. For example, if the purpose of AOP development is focused on development of viable alternative tests and end points, drilling down to the level of a molecular initiating event may not be needed if one can develop the AOP far enough to demonstrate the predictive utility of an alternative end point. In contrast, if the goal is to define chemical structure domains associated with the AOP (e.g., to support development of quantitative structure–activity relationship or read-across models), this will nearly always require resolution of the molecular initiating event [4].

In the case of swim bladder inflation, as we tried to link the organ-level key event to end points that could be measured in either an embryo test or in vitro, two types of conceptual models were needed based on the biological processes involved. Initial inflation of the swim bladder in most larval fish requires gulping of air [38]. This inflation occurs during a finite period of development bounded by formation of the pyloric sphincter, which creates a barrier between the bile and pneumatic ducts [38,40]. It was hypothesized that there were 2 general ways in which chemicals could interact with biological pathways to disrupt swim bladder inflation. The first was through disruption of the normal development and organization of the swim bladder and associated accessories, like the pneumatic duct, as a functional organ. The second was through disruption of normal functioning of that organ once formed. The cell types, biological pathways, and processes involved in each were sufficiently distinct that they were best represented in separate conceptual models (Supplemental Data, Figures S1 and S2).

Development and organization of the swim bladder and the molecular/biochemical signaling pathways involved have been studied in detail, particularly in zebrafish [33,41–43]. Briefly, under control of Wnt/β-catenin signaling, the swim bladder originates as an evagination of the foregut epithelium [43]. The epithelium elongates into a sac-like structure and pneumatic duct, while sonic hedgehog and indian hedgehog signaling coordinate the proliferation and organization of 2 additional layers of cells—a mesenchymal layer, which differentiates into smooth muscle under control of sonic hedgehog, and an outer mesothelial layer [41]. Endothelial cells associated with blood flow from capillaries surrounding the developing swim bladder also play a critical role in the later stages of epithelial growth, differentiation of the mesenchyme, and organization of the outer mesothelium [44]. At an even finer resolution, examining a conceptual model of the Wnt/β-catenin signaling pathway [45], one can start to identify specific molecular targets that contaminants like small organic molecules could potentially interact with. For example, exposure to inhibitors of the enzyme glycogen synthase kinase 3β can lead to β-catenin accumulation [46] and disrupted Wnt signaling. Therefore, glycogen synthase kinase 3β inhibition is a plausible molecular initiating event through which disrupted swim bladder development could manifest (Figure 4).

**Figure 5:**
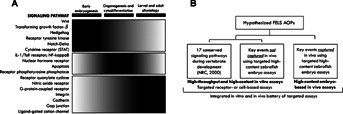
(**A**) Seventeen conserved signaling pathways identified by the National Research Council (NRC) and organized by stage of early vertebrate development. (**B**) Framework for development of targeted high-throughput screening and high-content screening for quantitative fish early life stage (FELS) adverse outcome pathway (AOP) discovery and annotation.

Other targets within the Wnt/β-catenin or hedgehog signaling pathways could be examined for linkage to the hypothesized AOPs for FELS mortality mediated via disruption of swim bladder inflation (Supplemental Data, Figure S3). However, it should be noted that involvement of these pathways in organogenesis is not unique to the swim bladder. Both Wnt and hedgehog signaling are among 17 signaling pathways identified as being highly conserved during development [47] (Figure 5A). Therefore, chemical disruption of these pathways would likely manifest as other effects that could be detected either in a fish embryo test or in molecular screening assays. For example, exposure of zebrafish embryos to certain polycyclic aromatic hydrocarbons and dibutyl phthalate has been shown to cause ectopic nuclear accumulation of β-catenin and associated disruption of dorsal–ventral patterning, a developmental process that occurs well before swim bladder formation [46]. Similarly, although the developing vasculature is critical for swim bladder development, the same could be said of many other essential organs in developing fish larvae. Therefore, development of AOPs linking disruption of these highly conserved pathways and processes to the key event of impaired swim bladder formation may not be critical, given the large number of other developmental events that would be impacted and the likelihood that such disruptions would be lethal and detected in an embryo assay.

We are aware of a single report in the literature that suggests the potential for specific effects of a chemical on swim bladder organogenesis, without any other observed toxic effects [35]. The authors hypothesize that tris(2,3-dibromopropyl)isocyanurate (TBC)-induced impairment of swim bladder formation may be a result of impaired secretion of surfactant mucus by the gas gland cells [35]. This mucus is similar to that found in the lungs of air-breathing vertebrates and is thought to function as an antiadherent that facilitates inflation and deflation of the organ [48]. However, it was not clear why TBC targeted gas gland cells without affecting other cell types [35]. Thus, there are important gaps in the hypothesized AOP associated with these observations (Figure 4).

A second conceptual model related to regulation of swim bladder function is presented in Supplemental Data, Figure S2. Once the swim bladder is formed and following initial inflation, gas exchange is largely regulated by diffusion of gas from the blood into and out of the swim bladder lumen [49]. In physoclists, whose pneumatic ducts degenerate following initial inflation, this is the only means for controlling inflation [38]. Physostomes maintain the capacity to transfer gas through the pneumatic duct, but most also transfer gas between the swim bladder and bloodstream [50]. Movement of oxygen into the swim bladder lumen, against a concentration gradient, is facilitated by lactic acid secretion from gas gland cells [51]. Lactic acid reduces the pH of the blood within the capillary network proximal to the gas gland cells, promoting release of oxygen from the hemoglobin as a result of the root effect [49,51]. This process involves a metabolic futile cycle of anaerobic glycolysis within the gas gland cells, suggesting that inhibition of either glucose transport (e.g., by glucose transporter 1a or 6 [glut1a, glut6]) or key enzymes like phosphofructokinase 1, fructose 1,6-bisphosphatase, or glyceraldehyde-3-phosphate dehydrogenase could lead to functional impairment of gas exchange [51] (Supplemental Data, Figure S4). Secretion of lactic acid from the gas gland cells and recycling from arterial capillary endothelial cells of the rete mirabile, mediated by specific solute transporters, are important regulators of the spatial localization of blood acidification [52]. Thus, these transporters are also potential molecular targets for chemicals that could potentially impair swim bladder function (Supplemental Data, Figure S4). Inflation and deflation of the swim bladder are also regulated by parasympathetic and adrenergic neurotransmission [50]. Consequently, neurotoxicants, like acetylcholinesterase inhibitors, would be hypothesized to potentially disrupt the normal regulation of swim bladder inflation. While this does not represent an exhaustive list of the potential molecular initiating events that could be linked to the key event of impaired swim bladder inflation, it provides an example of the general process that one can go through in trying to establish and evaluate plausible links in a hypothesized AOP.

As in the case of Wnt and hedgehog signaling and their roles in swim bladder development, enzymes involved in anaerobic glycolysis and parasympathetic neurotransmission have important functions in many cell types and tissues. Therefore, the specificity of any of these molecular initiating events for leading to impairment in swim bladder inflation versus a host of other effects that may occur is questionable. This question of specificity highlights the important role that assessing hypothesized AOPs according to the Bradford-Hill criteria has to play in the process of AOP development. Out of myriad putative AOPs that could be developed linking specific molecular initiating events to young-of-year mortality via impacts on swim bladder formation and maintenance, relatively few of those would be necessary to be adequately predictive and protective relative to the apical toxicological outcome.

## GAP FILLING/TARGETED TESTING

Identification of knowledge gaps within hypothesized AOPs is an expected outcome of the linkage evaluation process described above (Figure 1). Assuming there is sufficient reason to believe that a putative AOP may be of importance in terms of its specificity and toxicological relevance, the next logical step in AOP development would be to conduct targeted research to both fill gaps and address uncertainties in the putative AOP(s), if needed. In the TBC example above (Figure 4B), this would entail additional research to uncover the specific molecular target of TBC within the gas gland cells. If that molecular target is not specific to gas gland cells, gap filling may also require an understanding of exposure characteristics that make the gas gland cells particularly vulnerable to TBC, and whether those same characteristics would apply for other chemicals. The specific research questions will vary with the nature of the AOPs and gaps. However, developing a hypothesized AOP and conducting the linkage evaluation can help focus resources and research efforts on key questions that need to be addressed to facilitate making predictions based on an understanding of the pathway.

## AOP CATALOGING

The ultimate goal of AOP development is to document and catalog information that supports scientifically credible predictive relationships between measurable key events (Figure 1), facilitating extrapolation to the outcomes of concern. Consequently, as AOPs—even partial AOPs with gaps—are developed, it is valuable to catalog the key events, relationships between them, and the supporting evidence for those relationships in a knowledge base. The OECD has developed a guidance document on developing and assessing AOPs [30] that details the type of content that should ideally be included in an AOP description. This includes key event descriptions, supporting evidence for the links between key events, and overall assessment of the weight of evidence and confidence of the AOP in accordance with the Bradford-Hill criteria. However, it is recognized that there are significant limitations to an approach involving generation of a separate document for each AOP. While independent documents may be well suited for detailed scientific and technical review of individual pathways, they are an inefficient method for compiling AOP knowledge, particularly in a collaborative environment, and do little to facilitate the conceptualization of overlaps (e.g., shared key events), connections, and potential interactions between pathways.

Consequently, a more dynamic and flexible knowledge-base environment for housing and cataloging AOP information is under development. In coordination with the OECD's Extended Advisory Group on Molecular Screening and Toxicogenomics, which currently oversees AOP development activities within the OECD [53], developers from the European Commission's Joint Research Center, the US Environmental Protection Agency's (USEPA's) Office of Research and Development, the US Army Corps of Engineers, the International Quantitative Structure Activity Relationship Foundation, and the University of Borgas are collaborating on an AOP knowledge base. The AOP knowledge base is being designed to store the same types of information that would be included in an AOP description document developed according to OECD guidance. However, it has several important added advantages. First, in terms of efficiency, key events may be shared by multiple AOPs. Key event pages developed in the knowledge base can be linked to multiple independent pathways, meaning that the information related to that key event (i.e., a description of the biology and approaches for measuring and observing the event) does not need to be entered every time a new pathway utilizing that key event is developed. Likewise, the weight of evidence linking any particular pair of key events does not need to be reconstructed each time that a pair of events is connected. Rather, information already in the knowledge base could be linked rapidly and automatically to the development of a pathway that contains a previously established link. Second, the knowledge base provides easy accessibility and searching capacity. It is intended that the knowledge base will be available over the Internet [54], making it possible for all users internationally to contribute via a common platform. Additionally, since each added pathway, molecular initiating event, adverse outcome, or key event page is added to an ordered drop-down list, users can easily search and view the content already in the knowledge base, helping to avoid needless duplication of effort but also allowing users to add information and evidence to existing pages. In that regard, the knowledge base also offers version control and tractability. All changes made to the contents are logged and can be traced. As the knowledge base develops, it is anticipated that visualization capabilities along with contextual annotations (i.e., level of biological organization, localization, taxonomic group, time to effect, sex, etc.) should allow users to more readily conceptualize interactions between AOPs in a transparent and computable manner through the informatics structure that supports network thinking and analysis. Such capabilities should both support regulatory applications and enhance scientific inquiry concerning complex toxicological outcomes.

## ASSAY ALIGNMENT

Once putative AOPs have been identified and critical gaps have been filled, at least in regard to biological plausibility, this information can be used to support alternative test development. This involves aligning key events in relevant AOPs with alternative assays or end points that could potentially be used to predict hazard (i.e., likelihood of young-of-year mortality for the swim bladder example). In addition to the pragmatic prioritization outlined above, priority might be given to the development of assays or end points associated with key events that are shared by many AOPs and thus represent multiple important hubs in a toxicological response network. Consequently, it is useful to construct and catalog as much AOP information as possible up front so that the knowledge base can be mined to identify important nodes around which assay development can be focused.

By definition, key events are measurable or observable phenomena. Therefore, at least in theory, assay or end point development should be feasible for any key event. In practice, however, many measurements made in a research context may not be sufficiently rapid, cost-effective, and/or transferable to be amenable for use in routine chemical screening or toxicity testing. For the purpose of developing alternatives to the FELS test, the primary interest is in identifying high-throughput in vitro or cell-free assays that would either cover highly conserved signaling pathways common to vertebrate development or be predictive of key events that would not be captured in a fish embryo bioassay (Figure 5B). Inasmuch as an optimized fish embryo test can be viewed as one of the most viable near-term alternatives to OECD test guideline 210, end points that could be easily incorporated and measured in the context of an optimized embryo test would also be desirable (Figure 5B).

Relative to the examples described above, assays aligned with a number of the key events described in the putative AOPs are already available and in use. For example, an assay for human glycogen synthase kinase 3β activity that we had plausibly linked to impaired swim bladder development is already included in the USEPA's Toxcast program [55]. Assays for cholinesterase activity are also included in Toxcast [56]. Although we did not develop the linkage between impaired vascular development and impaired swim bladder development at the level of specific molecular initiating events, a battery of Toxcast assays predictive of vascular disruption has been developed [57]. These assays may have utility for predicting adverse effects on the development of organs, such as the swim bladder, whose development and function rely on proper organization of the surrounding vascular structures. Again, one could raise the question of the specificity of these assays for predicting toxicity mediated specifically through the key event of impaired swim bladder development, but the examples demonstrate the overall principle of aligning key events with potential high-throughput assays.

## QUANTITATIVE APPLICATION

Descriptions of key events within an AOP and the weight of evidence supporting links between those key events have immediate utility in the area of hazard assessment and prioritization. In principle, if a chemical is shown to trigger the molecular initiating event or 1 or more of the intermediate key events along the pathway, one has a credible basis to conclude that, if the perturbation is sufficiently severe, a particular adverse outcome such as reduced probability of young-of-year survival will be observed. This level of information can help prioritize which chemicals among thousands should likely be subjected to more costly apical toxicity testing or higher tiers of assessment. However, many regulatory applications require not just the ability to determine whether an adverse outcome could plausibly occur but also the ability to define the probability that the adverse outcome will occur under specific perturbation conditions (e.g., concentration, duration, frequency of exposure). This requires quantitative understanding of the concentration–response relationships between different key events in the AOP. These may take the form of correlations between the magnitude of one response variable versus another [58,59], thresholds of response that must be achieved to trigger a downstream event (i.e., a point of departure), or mechanistically based computational models that rely on a key event measurement as input data and yield a predicted response for an event or outcome farther downstream along the pathway [60,61]. Regardless of the form, such quantitative relationships are intended to provide the basis for quantitative extrapolation of effects measured at a level of biological organization (typically lower) to the downstream impacts they may trigger either directly or indirectly (typically at higher levels of organization). However, quantitative predictions of this sort require that uncertainties associated with the extrapolations be understood and quantifiable as part of a probabilistic assessment.

## RESEARCH STRATEGY

The preceding sections describe a generalized strategy for AOP development and the application of that strategy to the specific challenge of assembling associated knowledge that supports development of lower-cost, higher-throughput, and less animal-intensive alternatives to the FELS test. We present hypothesized AOP development and linkage evaluation centered on the key events of swim bladder inflation as an example of the type of considerations and analysis that go into AOP development. However, the example presented herein only minimally addresses the overall scope of AOP development needed to support the goals of the Animal Alternatives in Environmental Risk Assessment Technical Committee. Based on discussions from the 2012 workshop and subsequent work by the technical committee, a number of key research objectives that will support and enhance the effort have been defined.

### Objective 1: Expand and disseminate a conceptual model of normal fish development

Over the past several years, there have been significant advances in our understanding of the molecular pathways and specific gene products necessary for development of different organ systems. Many of these advances have been made using the zebrafish model. However, ready application of this new information has been hampered by the lack of a conceptual model that brings together not only data on morphological landmarks but also emerging information concerning the molecular, biochemical, and cellular signaling that regulates morphogenesis and development in a way that is useful for AOP derivation. Therefore, an important need (and project objective) is to build a web-based, user-accessible conceptual model of normal fish development. Given the availability of information for zebrafish, we recommend that this phase rely primarily on the extensive zebrafish literature available through multiple sources (e.g., PubMed, ZFIN, Scopus, Web of Science).

We envision that development of a conceptual model will require 3 separate steps. First, a discrete number of developmental landmarks need to be defined to guide model development. Examples of landmarks include development of the central nervous system, cardiovascular system, liver, and kidney (for example, Figure 2). Second, the specific timing, molecular pathways, and gene products functionally required for each developmental landmark need to be identified by mining the peer-reviewed literature and available mutant, morpholino, and chemical screening data. Finally, a user-accessible, web-based tool needs to be assembled, ideally in collaboration with and hosted by ZFIN [62], the primary online zebrafish model organism database used around the world. As a web-based model accessible through ZFIN, this tool would be freely available to the research community, as well as provide data browsing capabilities (similar to the National Center for Biotechnology Information) that would enable exploration of genes based on curated expression, phenotype, and ontology. As the AOP knowledge base [54] described in the *AOP Cataloging* section develops, this conceptual model could also be linked as a source for a controlled vocabulary and common reference points for fish development-related key events in the AOP knowledge base.

### Objective 2: Coordinated development of putative AOPs

Using the conceptual model as a guide, the technical committee supports initiation of a coordinated, systematic effort to develop AOPs over the biological landscape represented in the model. Using the swim bladder example as a guide, members of the technical committee and other interested collaborators will develop putative AOPs using the various developmental landmarks represented in the model as an anchoring point. This research activity would initially focus on assembling biological plausibility and supporting evidence for pathways from the extant literature and identifying key gaps in the putative AOPs, which could serve as topics for future research. The putative AOPs developed will be evaluated in accordance with OECD guidance and the Bradford-Hill criteria, and where plausibility and/or weight of evidence are sufficient, complete or partial AOPs can be deposited into the nascent AOP knowledge base.

### Objective 3: Development of alternative assays

Constructed AOPs would serve as the basis to identify and develop a battery of targeted in vitro and in vivo high-throughput screening and high-content screening assays that capture suborganismal end points within fish embryos and larvae (Figure 2). On the one hand, we recommend that high-content, imaging-based zebrafish embryo assays be used to capture developmental landmarks and key events that are observable and measurable in vivo. However, for developmental landmarks and key events that are not easily observable or measurable using high-content in vivo assays (e.g., smaller organs such as the thyroid or pancreas), targeted receptor- and cell-based assays should be identified and/or developed to capture these developmental landmarks and key events within high-priority AOPs.

### Objective 4: Characterize phase I and II biotransformation in fish embryos

Although cell and embryo assays can capture key events critical for FELS AOPs, differences in xenobiotic biotransformation capabilities within these systems, compared with intact juvenile fish, leads to uncertainty about the potential to enhance or mitigate chemical toxicity. For example, recent studies have demonstrated that zebrafish embryos lack the ability to transform allyl alcohol to acrolein and, as a result, are several hundred–fold less sensitive to the chemical than adult fathead minnows [63]. As such, although the zebrafish liver is considered mature at 5 d postfertilization [64], the use of zebrafish embryos and larvae to predict chronic fish toxicity requires fundamental knowledge of biotransformation enzyme activity during early fish development. Therefore, there is a need, for example, to characterize the protein expression dynamics of phase I and II biotransformation enzymes in zebrafish during developmental through adult stages to provide a direct comparison of biotransformation potential between embryos, larvae, and adults. Likewise, to the extent possible, the biotransformation capabilities of in vitro systems developed to support FELS–related hazard prediction and risk assessment should also be characterized so that related uncertainties can be defined.

### Objective 5: Define concentration–response curves and develop quantitative AOPs using reference chemicals

Following development of in vitro and in vivo assays that capture developmental landmarks and key events relevant to early fish development, the long-term goal is to develop quantitative extrapolation models anchored to FELS AOPs using a set of reference chemicals. Candidate reference chemicals that represent a range of chemical classes, mechanisms of toxicity, and susceptibilities to biotransformation (including bioactivation) will need to be identified. We envision that identification of these reference chemicals would involve 2 steps. First, using literature and database searching, an initial list of reference chemicals should be identified based on chemical class (e.g., pharmaceuticals, metals); known or postulated mechanisms of toxicity; availability of fish-specific acute, early-life stage, and chronic toxicity data; and availability of information from relevant quantitative structure–activity relationship and mode of action classification models (e.g., the USEPA's Ecological Structure Activity Relationship [ECOSAR] and Assessment Tool for the Evaluation of Risk [ASTER] programs). From this initial list, a subset of reference chemicals should then be selected using the following criteria: high target selectivity, to avoid chemicals with multiple targets, in terms of assessing AOP concordance and assay specificity; moderate to high potency to minimize off-target, systemic effects that may occur at high concentrations; inclusion of chemicals requiring bioactivation for toxicity (e.g., allyl alcohol); and representation of chemicals with existing OECD test guideline 210 data.

Testing of these reference chemicals has the benefit of serving to either help support or reject hypothesized AOPs and the predictive relationships represented within them. Additionally, it will generate data to help define concentration–response relationships that can support quantitative extrapolation of data derived from alternative assays to the regulatory end point of interest (i.e., young-of-year survival). The mechanistic understanding depicted in the conceptual model developed as part of objective 1, along with the data generated using reference chemicals, should serve as a foundation for development of in silico toxicological prediction models that use alternative assay data as inputs.

## CONCLUSIONS

Development of AOP knowledge is viewed as a critical scientific activity that can support the development and application of alternative methods for assessing chemical hazards. A generalized strategy for AOP development involves: 1) defining the purpose and scope of the AOP development activity; 2) assembling a conceptual model of the known biology concerning the system of interest; 3) imposing pragmatic priorities when needed; 4) linking key events via biological plausibility and weight of evidence into hypothesized AOPs; 5) where necessary, conducting research to fill critical gaps; and 6) cataloging the assembled information and weight of evidence in a manner that supports use by the regulatory and research communities. Specific to the purpose of alternative assay development, aligning efficient and cost-effective assays and end points with key events and defining concentration–response relationships that support extrapolation among key events are also critical steps. From a pragmatic standpoint, in the case of AOPs for FELS toxicity, it is most practical to initiate AOP development from an intermediate biological event and refine linkages to lower levels of biological organization (i.e., in the direction of the molecular initiating event) only to the extent that is necessary to arrive at a key event measurement that can be made in a more cost-effective and efficient manner than in the current FELS test. Likewise, specificity and relative sensitivity of the collection of putative FELS AOPs is a key consideration in terms of where to invest in assay development. While dozens of putative AOPs could be developed connecting molecular initiating events to the adverse outcome via different sequences of key events, identifying those pathways likely to be most sensitive and of greatest toxicological relevance in terms of providing a foundation for alternative assay development is an important challenge. The process of hypothesized AOP development and linkage evaluation is not necessarily designed to answer that question, but the question of specificity is a central one as it pertains to utilizing AOP knowledge to develop an appropriate, cost-effective battery of alternative assays and end points to refine or replace whole-animal guideline toxicity tests. The research objectives outlined in the present study are intended to guide the HESI Animal Alternatives in Environmental Risk Assessment Technical Committee and the broader scientific community in a productive program of research that will move the regulatory ecotoxicology community away from reliance on the FELS test for predicting fish chronic toxicity in much the same way that the FELS test itself shifted the ecotoxicology community away from reliance on fish full life-cycle tests.

## SUPPLEMENTAL DATA

**Figures S1–S4.** (533 KB PDF).

## Supporting Information

All Supplemental Data may be found in the online version of this article.
