# Supplementary material for: INVESTIGATING ALTERNATIVES TO THE FISH EARLY-LIFE STAGE TEST: A STRATEGY FOR DISCOVERING AND ANNOTATING ADVERSE OUTCOME PATHWAYS FOR EARLY FISH DEVELOPMENT
Source: Environ Toxicol Chem. 2013 Oct 1;33(1):158–69. doi: 10.1002/etc.2403 (PMC4119008; doi:10.1002/etc.2403)

Supplementary Figure S1. Conceptual model of swimbladder development.

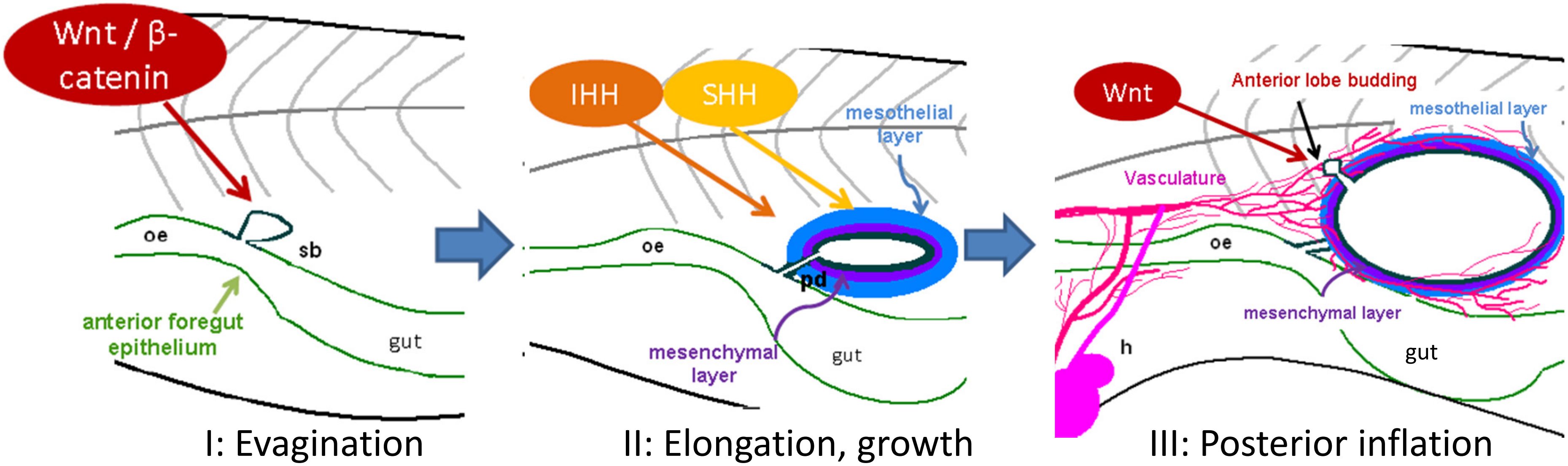

Adapted based on Winata et al. 2009.
oe = esophagus; sb = swimbladder; pd=pneumatic duct; h=heart; IHH=Indian hedgehog; SHH = sonic hedgehog.

Supplementary Figure S2. Conceptual model of key aspects of swimbladder function.

Inflation

•Parasympathetic acetylcholine-mediated neurotransmission

Deflation

• β-adrenergic receptor mediated neurotransmission

I. Passive diffusion (physotomes and physoclists)

I. Relaxation of smooth muscle, increased exposure of resorptive epithelium. (physotomes)

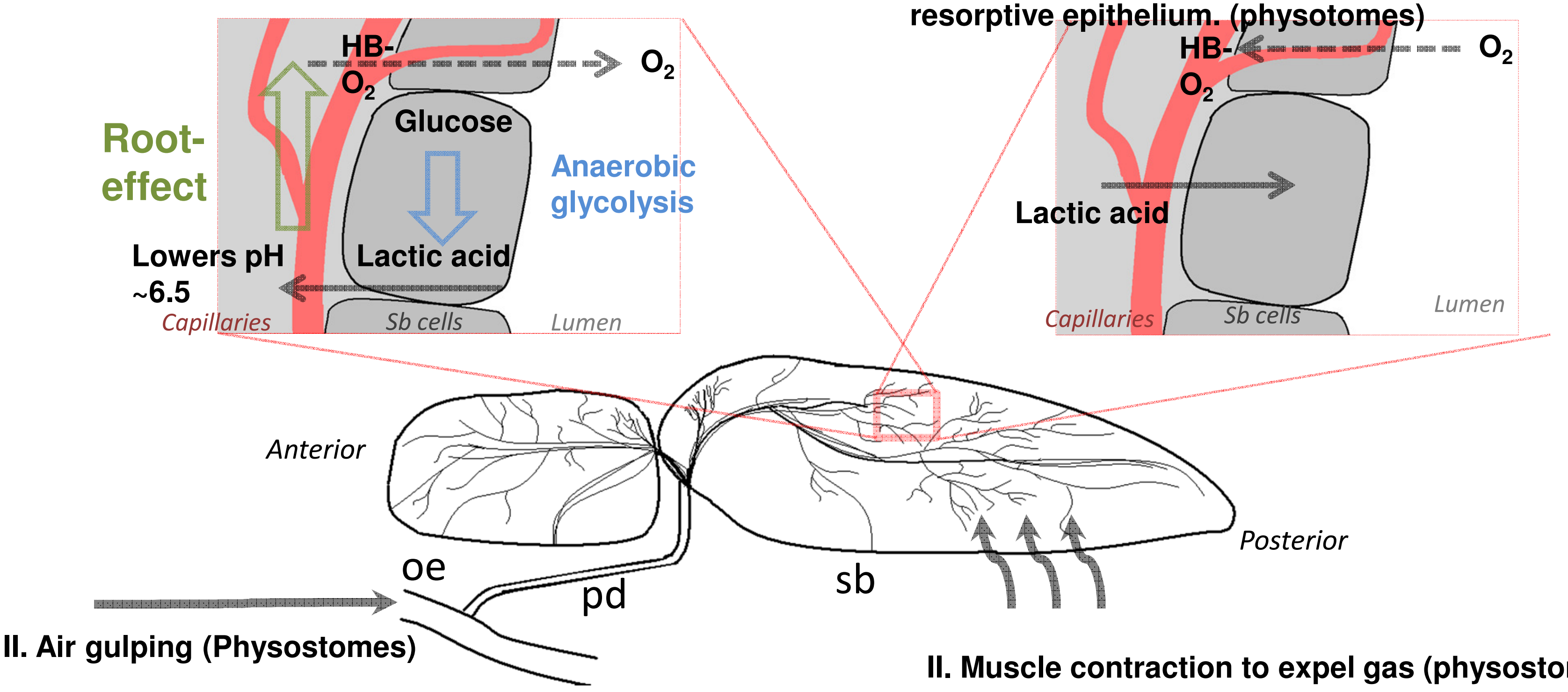

Adapted based on Dumbarton et al. 2010.
oe = esophagus; sb = swimbladder; pd=pneumatic duct

Supplementary Figure S3. Upstream biological perturbations during swimbladder formation plausibly linked to the key event of impaired swimbladder inflation.

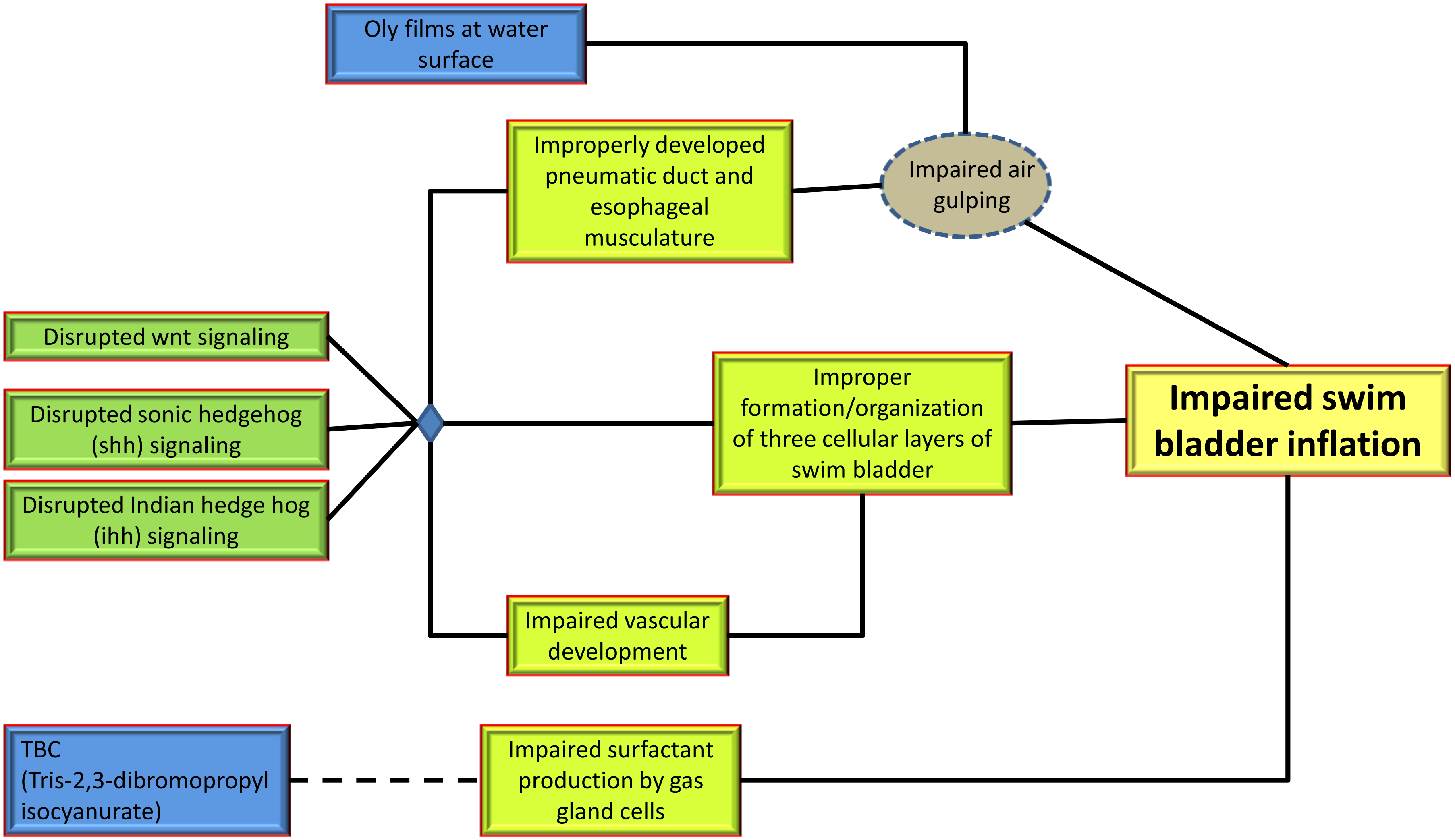

Supplementary Figure S4. Upstream biological perturbations plausibly linked to the key event of impaired swimbladder inflation.

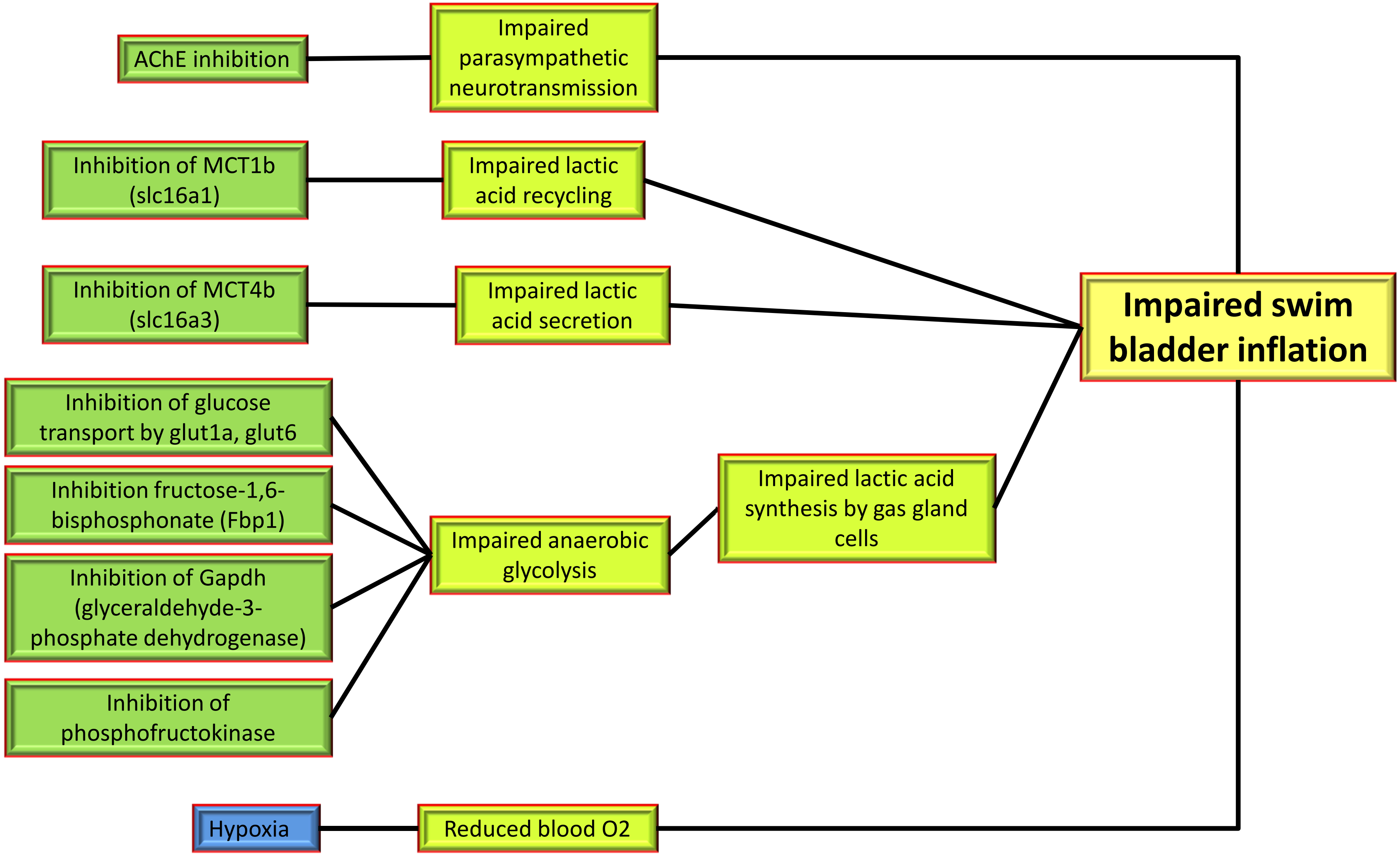

Supplement: Supplementary file 1 — Supplemental Data. [file etc0033-0158-sd1.pdf]
